# The Urban-Rural Disparities and Associated Factors of Health Care Utilization Among Cancer Patients in China

**DOI:** 10.3389/fpubh.2022.842837

**Published:** 2022-03-04

**Authors:** Haipeng Wang, Xingxing Hua, Nengliang Yao, Nan Zhang, Jialin Wang, Roger Anderson, Xiaojie Sun

**Affiliations:** ^1^Centre for Health Management and Policy Research, School of Public Health, Cheeloo College of Medicine (Key Lab of Health Economics and Policy, National Health Commission), Shandong University, Jinan, China; ^2^Affiliated Hospital of Integrated Traditional Chinese and Western Medicine, Nanjing University of Chinese Medicine, Nanjing, China; ^3^Department of Public Health Sciences, University of Virginia, Charlottesville, VA, United States; ^4^Center for Cancer Control and Policy Research, School of Public Health, Shandong University, Jinan, China; ^5^Shandong Academy of Medical Sciences, Shandong Cancer Hospital Affiliated to Shandong University, Jinan, China; ^6^University of Virginia Cancer Center, Charlottesville, VA, United States

**Keywords:** health care utilization, cancer patients, urban-rural disparities, associated factors, China

## Abstract

**Objective:**

This study aimed to examine the urban-rural disparities and associated factors of health care utilization among cancer patients in China.

**Methods:**

This study used the data collected from a cross-sectional survey conducted in China. A total of 1,570 cancer survivors from three urban districts and five rural counties were selected by using a multistage stratified random sampling method. We measured health care utilization with the way of cancer diagnosis, the number of hospitals visited, and receiving alternative therapies. Chi-square test was used to examine the differences between urban and rural cancer patients. Binary logistic regression analysis was performed to explore the determinants of health care utilization.

**Results:**

Among 1,570 participants, 84.1% were diagnosed with cancer after developing symptoms, 55.6% had visited two and above hospitals, and 5.7% had received alternative therapies. Compared with urban cancer patients, rural ones were more likely to be diagnosed with cancer after developing symptoms (χ2 = 40.04, *p* < 0.001), while they were less likely to visit more than one hospital (χ2 = 27.14, *p* < 0.001). Residence area (urban/rural), health insurance type, household income, age at diagnosis, tumor site, stage of tumor, and survival years were significantly associated with health care utilization of cancer patients (*p* < 0.01).

**Conclusions:**

Health care utilization was suboptimal among cancers patients in China. Rural cancer patients had less health care utilization including screenings and treatments than urban ones. Policymakers should implement specific strategies to ensure equitable utilization of cancer care. More attention should be paid to the disadvantaged groups and rural cancer patients. Prioritizing health resources allocation is needed to prevent, screen, and treat cancers in rural areas.

## Introduction

Globalcancer statistics estimated 18.1 million new cancer cases around the world in 2018, and out of these cases, nearly one-half occurred in Asia, 23.4% occurred in Europe, and 21% occurred in America. Thereinto, ~24% of cancer cases occurred in China (1). With the increasing incidence of cancer, the population burden of cancer is being more and more significant in China. Southwest China has the highest cancer incidence rate, followed by North China and Northwest China, and Central China has the lowest incidence rate (2). Cancer has become the leading cause of death (126.9 deaths per 100,000 persons) and a major economic burden in China (2, 3). In 2011, five types of most prevalent cancers (lung, stomach, colorectal, female breast, and esophageal cancers) accounted for 56% of the cancer burden in China (4). Improved detection methods and more effective therapies for cancer have been developed and applied in clinical practice, which has also led to the increasing number of cancer survivors (5, 6). Thus, cancer has become one of the most significant public health issues in China (7).

Timely and effective utilization of cancer care including screening, diagnosis, and treatment, is essential to the health outcome of cancer patients. It has been shown that medical detection and effective treatment are significantly associated with improved experiences and outcome with cancer survivorship (8, 9). Early medical screening can detect some types of cancers (breast, colorectal, and so on) before the symptoms start to show (10, 11). Even after initial treatment has been completed, cancer survivors may still need a spectrum of long-term following treatment and rehabilitation care, including alternative therapies (12). Cancer patients require a coordinated and multidisciplinary approach to treatment and control their illness (13), so they may need to visit more than one hospital for cancer care utilization.

Rural cancer survivors were considered as having higher risk for decrements in health and well-being due to decreased access to health care, specialty services, and support resources in the United States (14). Substantial urban-rural disparities in the likelihood of financial hardship and non-adherence to surveillance colonoscopy existed in New Mexico (15). In China, cancer patients living in rural areas have more restricted access to health care due to limited cancer care resources and shortage of qualified providers (16). Moreover, people living in rural China are also more likely to be diagnosed at more advanced stages of cancers (17). There is noticeable urban-rural inequality in access to health care and health resources in China. Urban residents are likely to have more healthcare utilization, more healthcare resources, more access to government sponsored public programs/healthcare services and less risk to suffer from diseases (18). Rural populations also have more restricted access to health care than urban residents due to low funds for health insurance scheme (19).

Health care utilization is the individuals' behaviors of choosing from a range of services and optional health care providers to meet their health demands (20). Health care utilization and its patterns are reflected by health care seeking behaviors (21, 22). Thus, health care can be well provided based on related information in terms of health care utilization and their associated factors (23). Many studies have used Andersen's behavioral models to examine the determinants of health care utilization, including predisposing factors, enabling factors, and need factors (23, 24). Existed literature has showed that health care utilization were affected by determinants at multiple levels, including individual and household factors (income, knowledge, education, age, gender, insurance coverage, distance to health facilities) (21, 25), as well as characteristics of health care system (availability, affordability and accessibility of drugs and healthcare services) (26). Furthermore, it has been revealed that urban-rural regional difference was also one important predictor of health care utilization (27).

Numerous studies have examined the incidence and mortality rates of cancer and their determining factors (2, 28). Also, many studies have highlighted issues related to cancer treatment, the cost of treatment, economic burden of cancer, and the lasting effects of treatment (29–31). Nevertheless, few studies have focused on cancer care utilization and its associated factors, especially among cancer survivors in China. Reliable information on patterns and determining factors of health care utilization is required to provide further insight for policy makers to take measures for cancer control and reduce the heavy burdens of cancers in China. Therefore, this study aimed to examine the urban-rural disparities and associated factors of health care utilization among cancer patients in China.

## Materials and Methods

### Data and Sample

This study used the data collected from a cross-sectional survey conducted from March 2015 to March 2016 in Shandong province of China, which used a modified questionnaire referred to the Cancer Supplement of the Medical Expenditure Panel Survey (32). A multistage stratified random sampling method was used in this survey. In the first stage, three urban districts and five rural counties were selected based on demographic context and socioeconomic development. The sample was stratified by rural vs. urban status. Counties and urban districts are at the same level in the Chinese administrative division system (32).

The survey selected patients with cancer identified in the cancer registry system as participants. The inclusion criteria of eligible participants were: (1) diagnosed with lung, stomach, colorectal, or female breast cancers between 2011 and 2013; (2) at least 18 years old at the time of their cancer diagnosis; and (3) having willingness to participate in the survey. Those patients were excluded if they were diagnosed with multiple cancers (33). In each county or district, about 200 eligible cancer patients were randomly chosen to complete the questionnaire by face-to-face interview. Finally, the survey assigned 1,600 cancer cases to interviewers, while a total of 1,570 cancer survivors were included in the final analysis of this study.

### Variables and Measurement

We measured health care utilization with the way of cancer diagnosis, the number of hospitals visited, and receiving alternative therapies. The three dependent variables were selected based on previous literatures and the profile of health care utilization among cancer patients in Chinese community. The way of cancer diagnosis can help us to provide evidence on the status of early detection of cancer. The number of hospitals visited can help us to understand the twists and turns during the cancer patients' diagnoses and treatments. The variable of receiving alternative therapies is lack of analysis in the majority of literatures, but alternative therapies are helpful for rehabilitation of cancer patients. The way of cancer diagnosis was measured based on the question: “How was your cancer diagnosed,” with answers (1, health checkup; 2, after developing symptom; 3, by testing for other illness; 4, cancer screening). The number of hospitals visited was the total number of visiting hospitals for the participant's treatment since diagnosed with cancer. Receiving alternative therapies was measured based on the question: “Have you tried alternative therapies besides hospital medical treatment.” To explore the influencing factors of cancer care utilization using logistic regression models, those dependent variables were all collapsed into binary variables, including the way of cancer diagnosis (1, after developing symptom; 0, others), the number of hospitals visited (1, more than one hospital; 0, one hospital), receiving alternative therapies (1, yes; 0, no).

According to the Andersen behavior model, the determinants influencing health care utilization consisted of predisposing factors, enabling factors and need factors (34). Predisposing factors included gender (1, female; 2, male), age at diagnosis (1, <50 year; 2, 50–59 year; 3, 60–69 year; 4, ≥70 year), educational level (1, < primary school; 2, primary school; 3, middle school; 4, ≥high school), marital status (1, married; 2, single/divorced/widowed), and employment status (1, employee; 2, farmer; 3, retired; 4, unemployed). Enabling factors included residence area (1, urban community; 2, rural village), health insurance type (1, UEBMI = Urban Employee Basic Medical Insurance; 2, URBMI = Urban Resident Basic Medical Insurance; 3, NCMS=New Cooperative Medical Scheme; 4, other), and annual household income (CNY) (1, <5000; 2, 5,000–20,000; 3, 20,000–50,000; 4, ≥50,000). Need variables included tumor site (1, breast; 2, lung; 3, colorectal; 4, stomach), cancer stage (1, early; 2, medium; 3, later; 4, unknown), and survival time (years) (1, <1 year; 2, 1–2 year; 3, 2–3 year; 4, ≥3year).

### Statistical Analysis

The hypotheses to be tested in this study are that there are significant disparities of cancer care utilizations (in terms of way of cancer diagnosis, number of hospitals visited and receiving alternative therapies) between urban rural areas, and many associated factors influencing health care utilization among cancer patients in China. Firstly, descriptive analysis was conducted to summarize basic characteristics and clinical characteristics of the respondents. The frequency and percentage were calculated and presented for categorical variables. Chi-square (χ2) test was used to examine the differences of proportions between urban and rural areas. Secondly, we used descriptive analysis to examine the frequency and percentage of health care utilization among the selected respondents. Chi-square (χ2) test was used to examine the differences of proportions between urban and rural cancer patients. Thirdly, binary logistic regression analysis was performed to explore the determinants of health care utilization (being diagnosed after developing symptom, visiting more than one hospital, receiving alternative therapies). We presented Odds Ratios (ORs) and their 95% Confidence Intervals (CIs). All statistical analyses were conducted using STATA 15. The significance level for statistics was set at *p* < 0.05 and *p* < 0.01.

## Results

### Basic Characteristics of The Participants and Urban-Rural Disparities

Basic characteristics were listed for the overall sample and by urban and rural participants (Table 1). Among 1,570 cancer patients, 983 (62.6%) were from rural areas. The majorities of them were females (56.1%), more than 50 years at diagnosis (78.5%), married (89.0%), farmers (64.3%), and covered by NCMS (67.4%). Only 17.1% of the participants had the education level of high school and above. More than half of the respondents had a low annual household income of <20,000 CNY. Cancer sites of the patients included breast (32.3%), stomach (26.0%), colorectal (24.8%), and lung (16.9%). More than a half of them had lived more than 2 years since their cancer diagnosis. Compared with urban cancer patients, rural ones were more likely to have low educational level (χ2 = 194.81, *p* < 0.001) and low household income (χ2 = 174.58, *p* < 0.001), to be at later cancer stage (χ2 = 17.79, *p* < 0.001), and to survive more than 2 years (χ2 = 58.48, *p* < 0.001). There were also statistical differences of gender, age at diagnosis, employment status and tumor site between urban and rural participants (all *p* < 0.05).

**Table 1:** Basic characteristics of the participants and urban-rural disparities (N, %).

	**Total (1,572)**	**Urban (587)**	**Rural (983)**	**χ^2^**	***P***
Gender					
Male	690 (43.9%)	225 (38.3%)	465 (47.3%)	12.01	**<0.001**
Female	880 (56.1%)	362 (61.7%)	518 (52.7%)		
Age at diagnosis (year)					
<50	338 (21.5%)	114 (19.4%)	224 (22.8%)	7.92	**0.048**
50–59	421 (26.8%)	162 (27.6%)	259 (26.3%)		
60–69	515 (32.8%)	182 (31.0%)	333 (33.9%)		
≥70	296 (18.9%)	129 (22.0%)	167 (17.0%)		
Educational level					
< Primary school	269 (17.1%)	87 (14.8%)	182 (18.5%)	194.81	**<0.001**
Primary school	544 (34.6%)	146 (24.9%)	398 (40.5%)		
Middle school	489 (31.1%)	154 (26.2%)	335 (34.1%)		
≥High school	268 (17.1%)	200 (34.1%)	68 (6.9%)		
Marital status					
Married	1,397 (89.0%)	514 (87.6%)	883 (89.8%)	1.92	0.166
Single/Divorce/Widowed	173 (11.0%)	73 (12.4%)	100 (10.2%)		
Employment status					
Employee	157 (10.0%)	91 (15.5%)	66 (6.7%)	460.46	**<0.001**
Farmer	1,009 (64.3%)	184 (31.3%)	825 (83.9%)		
Retired	255 (16.2%)	198 (33.7%)	57 (5.8%)		
Unemployed	149 (9.5%)	114 (19.4%)	35 (3.6%)		
Health insurance					
UEMI	280 (17.8%)	228 (38.8%)	52 (5.3%)	597.33	**<0.001**
URMI	166 (10.6%)	130 (22.1%)	36 (3.7%)		
NCMS	1,058 (67.4%)	176 (30.0%)	882 (89.7%)		
Other	66 (4.2%)	53 (9.0%)	13 (1.3%)		
Household income (CNY)					
<5,000	241 (15.4%)	58 (9.9%)	183 (18.6%)	174.58	**<0.001**
5,000–2,0000	562 (35.8%)	144 (24.5%)	418 (42.5%)		
20,000–50,000	522 (33.2%)	210 (35.8%)	312 (31.7%)		
≥50,000	245 (15.6%)	175 (29.8%)	70 (7.1%)		
Tumor site					
Breast	507 (32.3%)	247 (42.1%)	260 (26.4%)	43.75	**<0.001**
Lung	265 (16.9%)	91 (15.5%)	174 (17.7%)		
Colorectal	390 (24.8%)	131 (22.3%)	259 (26.3%)		
Stomach	408 (26.0%)	118 (20.1%)	290 (29.5%)		
Cancer stage					
Early	641 (40.8%)	260 (44.3%)	381 (38.8%)	17.79	**<0.001**
Medium	583 (37.1%)	231 (39.4%)	352 (35.8%)		
Later	137 (8.7%)	39 (6.6%)	98 (10.0%)		
Unknown	209 (13.3%)	57 (9.7%)	152 (15.5%)		
Survival time (year)					
1~2	739 (47.1%)	347 (59.1%)	392 (39.9%)	58.48	**<0.001**
2~3	456 (29.0%)	118 (20.1%)	338 (34.4%)		
3~4	375 (23.9%)	122 (20.8%)	253 (25.7%)		

*P-value from chi-squared test. UEBMI, Urban Employee Basic Medical Insurance; URBMI, Urban Resident Basic Medical Insurance; NCMS, New Cooperative Medical Scheme*.

### The Urban-Rural Disparity of Health Care Seeking Behaviors

Table 2 presented the urban-rural disparities of health care utilization of the participants. Among 1,570 cancer patients, 84.1% were diagnosed with cancer after developing symptoms. Approximately 43.3% of the participants had visited two hospitals for treatments, while 12.3% had visited three and above hospitals. Only 5.7% of them had received alternative therapies for cancer. Compared with urban cancer patients, rural ones were more likely to be diagnosed with cancer after developing symptom (χ2 = 40.04, *p* < 0.001), while were less likely to visit more than one hospital (χ2 = 27.14, *p* < 0.001).

**Table 2:** Health care utilization and urban-rural disparities among cancer patients (N, %).

	**Total**	**Urban**	**Rural**	**χ^2^**	***P*-value**
Way of cancer diagnosis					
Health checkup	114 (7.3%)	69 (11.8%)	45 (4.6%)	40.04	**<0.001**
After developing symptom	1,321 (84.1%)	452 (77.0%)	869 (88.4%)		
By testing for other illness	67 (4.3%)	33 (5.6%)	34 (3.5%)		
Cancer screening	28 (1.8%)	12 (2.0%)	16 (1.6%)		
Others	40 (2.5%)	21 (3.6%)	19 (1.9%)		
Number of hospitals visited					
1	697 (44.4%)	211 (36.0%)	486 (49.4%)	27.14	**<0.001**
2	680 (43.3%)	292 (49.7%)	388 (39.5%)		
≥3	193 (12.3%)	84 (14.3%)	109 (11.1%)		
Receiving alternative therapies					
No	1,480 (94.3%)	546 (93.0%)	934 (95.0%)	2.72	0.099
Yes	90 (5.7%)	41 (7.0%)	49 (5.0%)		

*P-value from chi-squared test*.

### Determinants of Health Care Utilization by Logistic Regression Models

Table 3 displayed the determinants of health care utilization by binary logistic regression analyses. Older cancer patients were less likely to visit more than one hospital (OR = 0.66, *p* < 0.05; OR = 0.53, *p* < 0.01). Compared with cancer patients having UEMI, those having URMI or NCMS were less likely to be diagnosed with cancer after developing symptoms (OR = 0.43, *p* < 0.01; OR = 0.48, *p* < 0.05). Cancer patients having higher household income were less likely to be diagnosed with cancer after developing symptoms (OR = 0.53, *p* < 0.05; OR = 0.54, *p* < 0.05; OR = 0.48, *p* < 0.05), while were more likely to visit more than one hospitals (OR = 2.02, *p* < 0.01). Compared with urban cancer patients, rural ones were more likely to be diagnosed with cancer after developing symptom (OR = 1.87, *p* < 0.01), but were less likely to visit two and above hospitals (OR = 0.59, *p* < 0.01).

**Table 3:** Determinants of health care utilization by binary logistic regression (OR, 95%CI).

	**Diagnosed after developing symptom**	**Visiting more than one hospital**	**Receiving alternative therapies**
Gender (ref. =Female)			
Male	1.12 (0.75, 1.68)	1.00 (0.76, 1.31)	1.06 (0.59, 1.91)
Age at diagnosis (ref. = <50)			
50–59	1.26 (0.81, 1.98)	0.85 (0.62, 1.17)	1.40 (0.73, 2.69)
60–69	0.94 (0.58, 1.51)	0.66 (0.47, 0.93)^*^	1.10 (0.52, 2.32)
≥70	0.98 (0.54, 1.80)	0.53 (0.35, 0.81)^**^	1.68 (0.68, 4.13)
Education (ref. = < Primary school)			
Primary school	1.40 (0.89, 2.20)	0.75 (0.55, 1.03)	1.00 (0.49, 2.22)
Middle school	1.65 (0.99, 2.73)	0.98 (0.69, 1.40)	1.60 (0.70, 3.66)
≥High school	0.84 (0.46, 1.53)	1.15 (0.72, 1.82)	2.37 (0.90, 6.24)
Marital status (ref. = Married)			
Single/Divorced/Widowed	1.01 (0.60, 1.70)	1.34 (0.93, 1.93)	0.84 (0.35, 1.99)
Working status (ref.= Employee)			
Farmer	1.58 (0.89, 2.80)	1.26 (0.81, 1.95)	0.53 (0.23, 1.23)
Retired	1.28 (0.70, 2.35)	1.41 (0.86, 2.32)	0.83 (0.35, 1.92)
Unemployed	1.66 (0.85, 3.24)	0.85 (0.50, 1.44)	0.14 (0.03, 0.71)^*^
Health insurance (ref.= UEMI)			
URMI	0.43 (0.24, 0.80)^**^	1.04 (0.64, 1.67)	1.01 (0.39, 2.65)
NCMS	0.48 (0.26, 0.90)^*^	1.26 (0.80, 1.99)	1.08 (0.44, 2.67)
Other	0.36 (0.19, 0.67)^**^	1.38 (0.74, 2.58)	1.46 (0.59, 3.62)
Household income (ref. = <5,000)			
5,000–20,000	0.53 (0.30, 0.92)^*^	0.89 (0.64, 1.23)	2.04 (0.93, 4.47)
20,000-50,000	0.54 (0.31, 0.94)^*^	1.07 (0.76, 1.50)	0.68 (0.28, 1.65)
≥50,000	0.48 (0.25, 0.91)^*^	2.02 (1.30, 3.14)^**^	0.90 (0.34, 2.35)
Location of tumor (ref. = Breast)			
Lung	0.81 (0.49, 1.31)	1.55 (1.08, 2.24)^*^	0.97 (0.46, 2.08)
Colorectal	1.65 (1.00, 2.71)^*^	0.87 (0.63, 1.21)	1.12 (0.57, 2.23)
Stomach	1.21 (0.73, 2.00)	1.09 (0.77, 1.54)	0.58 (0.26, 1.28)
Stage of tumor (ref.= Early)			
Medium	1.43 (1.02, 2.01)^*^	1.64 (1.29, 2.08)^**^	1.53 (0.91, 2.59)
Later	1.61 (0.89, 2.92)	1.49 (1.00, 2.20)^*^	2.20 (1.06, 4.56)^*^
Unknown	1.51 (0.93, 2.48)	0.93 (0.67, 1.29)	1.33 (0.65, 2.74)
Survival years (ref. = 1~2)			
2~3	1.19 (0.83, 1.72)	1.17 (0.91, 1.50)	1.67 (1.00, 2.80) ^*^
3~4	1.17 (0.81, 1.71)	0.89 (0.68, 1.15)	1.15 (0.65, 2.05)
Residence area (ref. = Urban)			
Rural	1.87 (1.26, 2.78)^**^	0.59 (0.44, 0.79)^**^	0.76 (0.41, 1.39)
Constant	4.67 (1.67, 13.06)^**^	1.21 (0.57, 2.53)	0.03 (0.01, 0.16)^**^
LR chi2	87.91	117.32	57.95
Prob	<0.001	<0.001	<0.001

*ORs, Odds ratios; 95%CI, 95% confidence intervals*.

* *P <0.05*;

** *P <0.01. UEBMI, Urban Employee Basic Medical Insurance; URBMI, Urban Resident Basic Medical Insurance; NCMS, New Cooperative Medical Scheme*.

Compared with breast cancer patients, lung cancer patients were more likely to visit more than one hospitals (OR = 1.55, *p* < 0.05); colorectal cancer patients were more likely to be diagnosed with cancer after developing symptoms (OR = 1.65, *p* < 0.05). Compared with cancer patients at early stage, those at medium stage were more likely to be diagnosed with cancer after developing symptoms (OR = 1.43, *p* < 0.05), to visit more than one hospitals (OR = 1.64, *p* < 0.01); and those at later stage, were more likely to visit more than one hospitals (OR = 1.49, *p* < 0.05) and to receive alternative therapies (OR = 2.20, *p* < 0.05). Compared with cancer patients surviving 1~2 years, those surviving 2~3 years were more likely to receive alternative therapies (OR = 1.67, *p* < 0.05).

## Discussion

This is the first study to provide a snapshot regarding the urban-rural disparity and determining factors of health care utilization among cancer patients in China. Information on the utilization of health care is essential to reform health policies based on the demands and influencing factors. The findings in this study revealed that, the majority of cancer patients were diagnosed after developing symptoms, and more than a half of them visited two and above hospitals for cancer treatments, while only a few of them received alternative therapies for cancer. Comparatively speaking, rural cancer patients had less health care utilization than urban ones. Health care utilization of cancer patients were mainly determined by both socioeconomic characteristics (health insurance types, household income) and clinical characteristics (age at diagnosis, tumor site, stage of tumor, survival years).

In the present study, 84.1% of the participants reported that they were diagnosed with cancer patients after developing symptoms, and only 13.4% of cancer patients were detected via health checkups, by testing for other illness, or tumor screening. Some reasons might explain this phenomenon: firstly, many of the respondents may have no adequate knowledge about cancer risk factors and symptoms. Cancer patients could not seek timely diagnoses at the specialized health institutions if they had no appropriate interpretation of early symptoms (35). Secondly, health checkups and cancer screening services have not been covered by social health insurance schemes for the general population in China (32), although several big public health programs have been implemented among high risk population. Therefore, promoting diagnoses at early stage is still a big challenge in China. On one hand, there is a big need of health education interventions to increase the cancer knowledge and diagnostic acumen of both healthcare professionals and high-risk population. On the other hand, if the benefit packages of social health insurance schemes can be expanded to cover some major cancer screening services, more cancer cases will be timely diagnosed and treated to reduce the incidence of cancer at advanced stages.

This study showed that 55.6% of participants had visited two or more hospitals for cancer treatments. After the detection of primary cancer signals, the patients should be referred to the specialized cancer facilities for further diagnosis and treatment. However, they often postpone health care utilization and miss appointments for their treatments due to lack of money or other reasons. Social health insurance should play a greater role in reducing out-of-pocket expenditure on healthcare services for cancer patients. Cancer patients usually use health care in specialized cancer treatment facilities. Specialized cancer treatment facilities that are closer to the patients would facilitate health care utilization and adherence to treatment regimens (35). Ongoing efforts should be taken to integrate cancer care between oncology and primary care settings. The study also found that only a few of them had received alternative therapies for cancer. Models for the integration of care for cancer survivors, including self-management, wellness and healthy lifestyle promotion, and cancer rehabilitation, should be developed and applied (6).

The findings indicated that there were differences in health care utilization between urban and rural cancer patients. Rural cancer patients were more likely to be diagnosed with cancer after developing symptoms and less likely to visit two or more hospitals than urban ones. This discrepancy is mainly because there are obvious disparities in healthcare resources including infrastructure and human resource between rural and urban areas (18). It was reported that in 2015, the number of health technicians and hospital beds per thousand persons in urban areas was nearly 2.62 times and 2.23 times more than those in rural areas in China, respectively (36). Therefore, patients in rural areas may have less access to specialist medical care, and this should be an important consideration when defining future healthcare investment and policy (37). It is vital to reduce the disparity of healthcare resources between rural and urban areas by improving the capacity of rural healthcare institutions in China (18). During the study period, rural residents were mostly insured by the New Rural Cooperative Medical System, which had less coverage and higher coinsurance rates than the insurance that covered urban employees and residents (19).

Furthermore, the results suggested that health care utilization of cancer patients were influenced by both socioeconomic characteristics and clinical characteristics. Consistent with previous studies, health care utilization were influenced by health insurance and household income (21, 38). Cancer survivors may be at greatest risks for financial hardship due to the high expenditures on health services and medicines for cancer. If the burden of financial hardship from cancer is not addressed, the consequent impact on medical care access and utilization may lead to poor health outcomes among individuals with poor socioeconomic status (38, 39). This study also found that health care utilization of cancer patients were affected by age at diagnosis, location of tumor, stage of tumor, and survival years. Understanding personal socioeconomic and clinical features can help to identify sub-groups of cancer survivors with barriers to receipt of necessary cancer care, and to develop potential intervention points that ensure access to care across populations (40).

This is the first representative study that examined the urban-rural disparity and associated factors of health care utilization among cancer patients in China. However, there are several limitations to acknowledge in this study. Firstly, given the cross-sectional survey, the present analysis cannot identify the causal relationship between the identified determinants and health care utilization. Further research should investigate the causality with longitudinal survey data. Secondly, although we explored a broad set of individual factors associated with health care utilization from the demand side, no related information on health care system (availability, affordability and accessibility of healthcare services) from the supply side which might also influence health care utilization were involved, which will be explored in future study. Thirdly, our data were collected using a structured interviewer-administered questionnaire based on self-reported information of the participants, which might be subjected to recall bias.

In conclusion, health care utilization was suboptimal among patients with cancers in China. There were evident disparities in health care utilization between urban and rural cancer survivors. Health care utilization was determined by health insurance types, household income and clinical characteristics. The findings highlighted the needs of systematic collection of information on health care utilization of cancer patients. This would help policymakers to implement specific strategies for ensuring equitable utilization of health care, and to enforce the sufficient delivery of cancer care services in the country. More attention should be paid to the disadvantaged groups and rural cancer patients. Prioritizing health resources allocation is needed to prevent, screen, and treat cancers in rural areas in the future.

## Data Availability Statement

The original contributions presented in the study are included in the article/supplementary material, further inquiries can be directed to the corresponding author.

## Ethics Statement

This study was reviewed and approved by the Ethics Review Board of the School of Public Health, Shandong University (No. 20140201). All included participants provided written informed consent forms for participation in the survey before starting the interviews.

## Author Contributions

HW and XS conceived and designed the study. XH and HW did the data analysis. HW wrote the first draft of the paper. XS supervised and revised the first draft. All authors reviewed and approved the final manuscript submitted for publication.

## Funding

This work was supported by the China Medical Board (CMB 13-160).

## Conflict of Interest

The authors declare that the research was conducted in the absence of any commercial or financial relationships that could be construed as a potential conflict of interest.

## Publisher's Note

All claims expressed in this article are solely those of the authors and do not necessarily represent those of their affiliated organizations, or those of the publisher, the editors and the reviewers. Any product that may be evaluated in this article, or claim that may be made by its manufacturer, is not guaranteed or endorsed by the publisher.
